# Reply to Alavian and Tabatabaei: Serum viral markers in Iranian patients with congenital bleeding disorder

**DOI:** 10.4103/0256-4947.55179

**Published:** 2009

**Authors:** Alireza Abdollahi

**Affiliations:** Department of Pathology, Tehran University of Medical Sciences, Imam Boulevard Ave. Tehran, 1446984313, Islamic Republic of Iran T: +982-18-826-9844 F: +982-18-827-7321 dr_p_abdollahi@yahoo.com

**To the Editor:** I appreciate the comments of Drs. Alavian and Tabatabaei on our article in the November-December 2008 issue of the *Annals*.^1^ The HCV positivity rate of 40% mentioned in previous studies is related to a survey of hemophilic children aged under 12. The survey was conducted in the Clinic for Hemophilic Children, Tehran, Iran. The individuals concerned have taken coagulation factors and products after virus inactivation (after 1985). The study carried out at Imam Khomeini Hospital Tehran, Iran, and involved nearly 400 of the total hemophilic patients (adults or children) admitted there with HCV positivity of 80% to 85%.^2^ The reason for the increase could be explained by the use of coagulation factors contaminated with pre-1985 HCV and the lower referral of hemophilic patients to Imam Khomeini Hospital due to the breakout of HIV and the patient's fear for other sources of infection. Hemophilic patients are exposed to the threat of infection with other viruses and society has to pay more attention to this undeniable fact.
